# Minerals and Human Health: From Deficiency to Toxicity

**DOI:** 10.3390/nu17030454

**Published:** 2025-01-26

**Authors:** Mohammed S. Razzaque, Sunil J. Wimalawansa

**Affiliations:** 1 Department of Medical Education, University of Texas, Rio Grande Valley, Edinburg, TX 78520, USA; msr.boston@gmail.com; 2 Cardiometabolic Institute, Brunswick, NJ 08902, USA

**Keywords:** biological functions, calcium, magnesium, micronutrients, public health, nutrients, physiology

## Abstract

Minerals are essential nutrients that play critical roles in human health by regulating various physiological functions. Examples include bone development, enzyme function, nerve signaling, and the immune response. Both the deficiencies and toxicities of minerals can have significant health implications. Deficiencies in macrominerals such as calcium, magnesium, and phosphate can lead to osteoporosis (associated with falls and fractures), cardiovascular events, and neuromuscular dysfunction. Trace mineral deficiencies, such as iron and zinc. Selenium deficiency impairs oxygen transport, immune function, and antioxidant defenses, contributing to anemia, delaying wound healing, and increasing susceptibility to infectious diseases. Conversely, excessive intake of minerals can have severe health consequences. Hypercalcemia can cause kidney stones and cardiac arrhythmias as well as soft-tissue calcification, whereas excessive iron deposition can lead to oxidative stress and organ/tissue damage. Maintaining adequate mineral levels through a balanced diet, guided supplementation, and monitoring at-risk populations is essential for good health and preventing disorders related to deficiencies and toxicities. Public health interventions and education about dietary sources of minerals are critical for minimizing health risks and ensuring optimal well-being across populations. While a comprehensive analysis of all macro and micronutrients is beyond the scope of this article, we have chosen to focus on calcium, magnesium, and phosphate. We summarize the consequences of deficiency and the adverse events associated with the overconsumption of other minerals.

## 1. Introduction

Adequate mineral balance is crucial for maintaining normal human health and preventing disease. Minerals, which are essential for sustaining various physiological functions, are classified into two main groups: (1) macrominerals, which primarily include calcium (Ca), phosphorus, magnesium (Mg), sodium, and potassium; and (2) microminerals (trace minerals), such as iron, zinc, copper, iodine, selenium, and manganese [1,2,3]. These minerals are vital for the growth and development of bones and teeth and the physiological functions of muscles and nerves. Additionally, minerals help maintain fluid balance, regulate pH levels, and serve as cofactors for numerous enzymes and coenzymes.

Specific mineral ions are involved in selective functions, ranging from exerting antioxidant effects (selenium, zinc, and copper) to hormone production (iodine for thyroid hormones). Iron deficiency is one of the most prevalent nutritional deficiencies worldwide; it affects more than 25% of the global population and is a major cause of anemia [4]. Vitamin D deficiency is also widespread, with approximately 23% of children in Africa estimated to have vitamin D deficiency [5]. Iodine deficiency can lead to an enlarged thyroid gland (goiter), fatigue, weight gain, dry skin, and impaired cognitive development in children.

**Figure 1:**
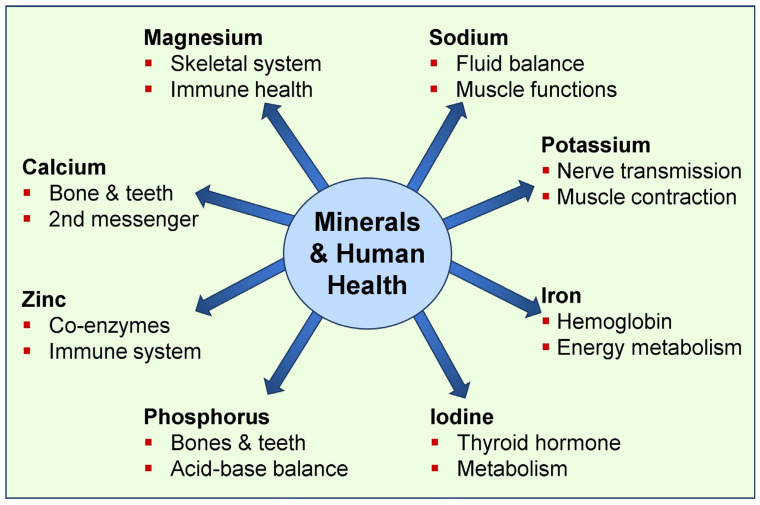
The figure depicts essential minerals with primary functions.

An adequate amount of zinc is essential for physiological cellular functions. It is a cofactor for more than 600 enzymes, facilitating DNA and RNA synthesis and playing critical roles in immune function and wound healing. Zinc is also involved in protein synthesis and cell division [1]. Zinc deficiency can slow wound healing, impair oral health [6], and lead to symptoms such as loss of taste or smell, hair loss, skin lesions, and diarrhea [7]. Selenium is an antioxidant in physiological amounts that protects cells from oxidative damage caused by reactive oxygen species (ROS) and reactive nitrogen species [8]. It also supports thyroid hormone metabolism and has immunoregulatory functions [9,10]. Figure 1 illustrates several essential minerals and their fundamental functions in humans.

This review focused on essential minerals Ca, Mg, and phosphate because of their physiological importance; they account for around 98% of the body’s mineral content by weight. More importantly, Ca, Mg, and phosphate have agonist–antagonist and interrelated metabolic pathways (see Section 2). For instance, parathyroid hormones regulate the levels of all three minerals, making their study as a group particularly relevant. The focused approach presented here comprehensively describes the most abundant and interconnected minerals while acknowledging the importance of other minerals in health and disease.

### 1.1. The Importance of Micro-Minerals in Physiological Functions

The physiological functions of iron vary from being an essential component of hemoglobin and myoglobin to being crucial for oxygen transport in the blood and energy metabolism (Table 1). Zinc, selenium, and iron inadequacy can impair immune responses and increase infection susceptibility [11]. Potassium and Mg help regulate blood pressure and heart rhythm, and the inadequacy of these minerals may increase the risk of hypertension and cardiovascular diseases. Below, we explain the clinical aspects of Ca, Mg, and phosphate and summarize the importance of other essential minerals for human health.

### 1.2. Broader Physiological Functions of Macro-Minerals

Physiological Mg balance is crucial for muscle contraction and nerve function. Mg also activates enzymes and supports intracellular signaling; it plays an essential role in DNA and RNA synthesis, stabilizing membrane structures and potential [1]. Mg deficiency can induce muscle cramps, twitches, fatigue, irregular heartbeats, osteoporosis, hypertension, and migraines [12,13]. In addition, it may contribute to steroidogenesis through its effects on enzyme activity and cellular signaling pathways [14,15].

Vitamin D is an essential micronutrient critical for regulating the metabolism of mineral ions, including Ca and phosphorus. It is also necessary for normal muscle function, bone health, and immune system regulation. Vitamin D deficiency can impair intestinal mineral ion absorption, renal mineral ion reabsorption, and bone resorption. Insufficient levels of vitamin D may lead to bone pain, muscle weakness, an increased risk of fractures, fatigue, and depression. These symptoms are mediated partially by Ca inadequacy. Ma and zinc are also required to properly process and activate vitamin D and its receptors [16,17].

Both mineral deficiency and excess can have adverse health consequences. For instance, the increased consumption of processed foods containing phosphate additives can lead to chronic phosphate toxicity, which may cause bone deformities, cardiovascular calcification, metabolic disorders, and accelerated aging [18,19,20,21,22]. While a balanced diet typically provides sufficient minerals, understanding their specific roles and ensuring adequate intakes are essential for overall well-being and disease prevention.

Notably, certain groups may be at risk of mineral deficiencies, including pregnant women, elderly individuals, vegetarians/vegans, people with certain medical conditions, and those following restrictive diets. In such cases, targeted supplementation under medical supervision may be necessary. Nutrient deficiencies manifest nonspecific symptoms so that blood levels may offer valuable insights into the underlying pathophysiology. Table 1 lists common minerals, their sources, and their physiological functions.

Most of the information in the Table was compiled from the Dietary Supplement of the Institute of Medicine and Fact Sheets and recommended daily allowances from the National Institute of Health (NIH), Office of Dietary Supplements (ODS) (https://ods.od.nih.gov/, Accessed on 3 January 2025).

## 2. Calcium—From Physiology to Pathology

Calcium (Ca) is essential for numerous physiological processes, including bone health, muscle contraction, nerve signaling, and blood clotting. Approximately 99% of the body’s Ca is stored in bones and teeth, providing structural support and serving as a reservoir to maintain extracellular Ca levels. Adequate Ca intake prevents osteoporosis and maintains bone density, particularly in aging populations and postmenopausal women [23]. Beyond skeletal health, Ca is a key regulator of cellular functions such as enzyme activity and signal transduction, emphasizing its importance in overall metabolic processes [24].

Normal Ca balance is essential for neuronal functions, cardiac activities, and blood coagulation [25,26]. Ca deficiency is common and can cause various symptoms, including numbness and tingling in the fingers and toes, muscle cramps, arrhythmia, bone deformities (osteoporosis), and fractures. C deficiency can also lead to serious health issues, such as cardiac arrhythmias, which may require immediate medical intervention [27]. In pregnant women, there is an increased risk of preeclampsia and related complications associated with insufficient Ca (and vitamin D) intake [28].

### 2.1. The Impact of Global Calcium Deficiency

**Figure 2:**
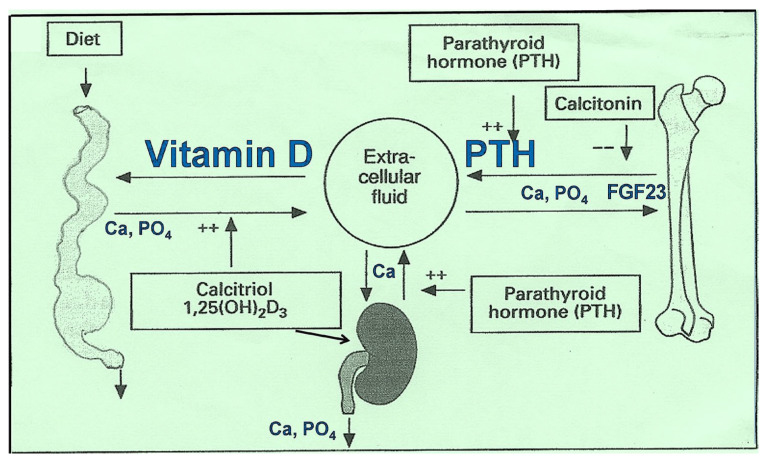
Major physiological and biological regulators and controls are illustrated [Fibroblast growth factor 23 (FGF23)].

An estimated 3.5 billion people worldwide are at risk of Ca deficiency due to inadequate dietary intake. Populations in low- and middle-income countries, particularly in parts of Asia, Africa, and South America, are at risk of low Ca intake. Conversely, people in Western countries have an excessive Ca intake. Consequently, they tend to have higher Ca:Mg increase risks for hypercalcemia, potentially leading to kidney stones, vascular calcification, and impaired renal function [29]. Balancing Ca levels through diet and supplementation, as needed, is essential for supporting long-term health and preventing complications. Foods such as dairy products, leafy greens, and fortified alternatives remain primary sources of dietary Ca, underscoring their accessibility and importance in public health initiatives. The primary physiological regulators of Ca metabolism and related mechanisms are illustrated in Figure 2.

### 2.2. Causes of Hypocalcemia

Causes of hypocalcemia are multi-factorial and include dietary insufficiencies, vitamin D deficiency, hypoparathyroidism (due to any case), and renal dysfunction. Acute symptomatic hypocalcemia presents with tetany, irritability, mental issues, or cardiac arrhythmias; these may require immediate attention with intravenous administration of Ca gluconate or Ca chloride to restore serum Ca levels [27]. Monitoring serum Ca, Mg, and phosphate levels ensures effective correction and prevents recurrence.

### 2.3. Treatment of Hypocalcemia

Hypocalcemia is managed by addressing the underlying causes, such as vitamin D deficiency, hypoparathyroidism, or renal dysfunction. Acute symptomatic hypocalcemia, which presents with tetany or cardiac arrhythmias, requires immediate intravenous administration of Ca gluconate or Ca chloride to restore serum Ca levels [27]. To increase intestinal Ca absorption, chronic hypocalcemia is typically treated with oral Ca supplements and active vitamin D, calcitriol or its synthetic analogs [27]. In addition, it can be treated with newer agents, like Yorvipath (palopegteriparatide) [30], to increase intestinal Ca absorption [27]. Monitoring serum Ca, Mg, and phosphate levels ensures effective correction and prevents recurrence [31].

### 2.4. Causes of Hypercalcemia

Hypercalcemia, characterized by elevated serum Ca levels, is most commonly caused by primary hyperparathyroidism and malignancies [32,33] that lead to increased bone resorption or ectopic production of parathyroid hormone (PTH)-related proteins [29,31]. Other causes include excessive Ca intake and/or vitamin D supplements (milk-alkali syndrome) and granulomatous diseases such as sarcoidosis, where activated macrophages produce excessive calcitriol [31,34]. Medications such as thiazide diuretics and lithium can also contribute to hypercalcemia by reducing Ca excretion or increasing parathyroid hormone (PTH) secretion.

### 2.5. Treatment of Hypercalcemia

Elevated serum Ca levels define hypercalcemia. Its treatment is based on the underlying etiology, development (acute vs. chronic), and severity, such as malignancy, hyperparathyroidism, or excessive Ca intake. Mild cases often require hydration and dietary modification to reduce Ca levels. More severe cases necessitate aggressive rehydration with intravenous saline to promote renal Ca excretion, followed by loop diuretics such as furosemide if needed. Bisphosphonates, such as zoledronic acid, effectively reduce bone resorption in hypercalcemia of malignancy [32], while calcitonin provides a rapid, albeit temporary, reduction in serum Ca [35]. In refractory or life-threatening cases, dialysis may be required to remove excess Ca, particularly in patients with renal impairment [32].

## 3. Magnesium—From Physiology to Pathology

Magnesium (Mg) is essential for many physiological functions, including enzymatic activity, hormone synthesis and release, neuromuscular function, cellular energy balance, and receptor activation [36,37]. Consequently, hypomagnesemia can have adverse effects on human health. Individuals may experience muscle weakness, cramps, tremors, and fatigue when Mg levels fall below the normal range [38]. Hypomagnesemia can also lead to cardiac arrhythmias, as Mg is crucial for maintaining proper heart rhythm and regulating potassium and Ca ions in cardiac tissues [39].

Chronic hypomagnesemia has been linked to a range of long-term health problems, including an increased risk of falls, osteoporosis, hypertension, and insulin resistance. Circulatory Mg concentrations do not accurately reflect tissue Mg levels [13]. Lower tissue Mg can lead to cardiac arrhythmia, nerve conduction defects, and muscle weakness [37]. It may also impair parathyroid gland function and exacerbate conditions such as diabetes and metabolic syndrome [39]. In severe cases, hypomagnesemia can lead to life-threatening complications, such as respiratory distress and seizures [40]. Early diagnosis and management, through dietary adjustments or supplementation, are crucial to prevent adverse outcomes and restore Mg balance in the body [31,34].

Hypomagnesemia also disrupts hormone release and vitamin D activity [41]. It is associated with decreased synthesis and activation of vitamin D and its receptor (CTR)interactions, increased oxidative stress, and exacerbated cytotoxic activity in T lymphocytes, promoting the cytokine storm [42,43]. In addition, hypomagnesemia causes abnormal platelet aggregation and coagulation abnormalities [44,45], damage to the myocardium [46], and endothelial dysfunction [47,48]. These compounds are present in severely ill COVID-19 patients and are associated with adverse effects related to COVID-19 vaccines [49,50]. In addition to vitamin D and Mg, there are other essential micro-nutrients. These include iodine, vitamins C and A, B_2_, B_6_, and vitamin B_12_, omega-3 fatty acids, zinc, selenium, copper, and folate. Most of the world’s population is deficient in several of these nutrients, thus increasing their vulnerability to diseases.

### 3.1. Effects of Hypermagnesemia

Hypermagnesemia can cause neuromuscular dysfunction, manifesting as lethargy, muscle weakness, diminished deep tendon reflexes, hypotension, nausea, facial flushing, ileus, and flaccid muscle paralysis, which can progress to paralysis in severe cases [38,51]. It can also lead to cardiovascular complications, including hypotension, bradycardia, and, in extreme cases, cardiac arrest due to the inhibitory effect of Mg on Ca -mediated cardiac conduction pathways [52].

Additionally, elevated Mg levels can depress central nervous system activity, leading to respiratory depression or coma [53]. Severe hypermagnesemia (>6.0 mg/dL) is a medical emergency that often requires interventions such as intravenous Ca gluconate and renal replacement therapy to reduce Mg levels rapidly [51]. Early recognition and prompt management are critical to prevent life-threatening outcomes [38].

### 3.2. Treatment of Hypo- and Hypermagnesemia

In clinical practice, the management of hypomagnesemia involves identifying and addressing the underlying causes, such as poor dietary intake, gastrointestinal losses, or renal waste [38]. Mild cases can typically be treated with oral Mg supplements, such as Mg oxide or citrate [41,48]. For more severe cases, particularly when symptoms like arrhythmias or seizures are present, intravenous Mg sulfate is administered to restore levels rapidly [38]. Monitoring serum Mg levels during treatment is essential to prevent overcorrection and avoid complications such as hypermagnesemia [37,48].

## 4. Effects of the Ca–Mg Balance in Physiology

In addition to bones and teeth, Ca is found mainly outside the cells, while Mg is primarily intracellular. Ca is involved in several pathophysiological aspects of the interaction between SARS-CoV-2 and human host cells [54,55]. Similar interactions between hypocalcemia and the severity and mortality of SARS-CoV-2 infection have also been reported [56,57]. In addition, low serum Ca (and low Mg) levels are considered prognostic factors for determining the severity of the disease [58] and eventual clinical outcomes [56,59].

### 4.1. Healthy Balance of Ca:Mg Ratio

The balance between Ca and Mg is crucial for maintaining proper physiological functions, as both minerals play key roles in cellular signaling, muscle contraction, nerve transmission, and cardiovascular health [48]. However, this is neglected in clinical practice. Most guidelines and recommendations do not even mention such. Ca and Mg interact to ensure proper neuromuscular function and cellular stability and play a role in aging [60]. Mg acts as a natural Ca blocker, helping to prevent excessive Ca influx into cells. Disruption of this balance can lead to a variety of health issues. For example, low Mg levels can increase Ca entry into cells, leading to muscle spasms, arrhythmias, and hypertension.

Conversely, elevated circulatory Ca levels can inhibit Mg actions, exacerbating symptoms of Mg deficiency [61]. The calculated Ca:Mg ratios from the diet and dietary supplements (total intake) would benefit from using Mg supplements to establish a favorable ratio to counter adverse effects. The data confirmed that a proper Ca:Mg ratio (another biomarker of health) could mitigate certain chronic diseases in the long term. The optimal intake of Ca:Mg and circulatory ratios is approximately 2 [62]. Either a high dietary Ca:Mg (>2.60) ratio, or a low intake ratio (<1.70) are unphysiological and increase health risks and disorders [63]. Therefore, maintaining the correct Ca to Mg ratio around 2.0 is necessary for optimal health [63].

Cellular influx/effluxes of Mg involve the same transporters as those for Ca; Mg acts physiologically to counter Ca [58,64,65,66,67]. Consequently, one needs to focus on the relationships of the serum Mg-to-Ca ratio with disease conditions and severity, including mortality from severe COVID-19 [58]. Mg is critical for inhibiting ROMK potassium channels in the principal cells of collecting tubules and ducts. When Mg levels decrease, ROMK channels become hyperactive. Consequently, hypomagnesemia can lead to hypokalemia [64,68].

### 4.2. Ca-to-Mg Ratio for Physiological Functions

Mg is essential for many enzymatic and B-vitamin functions, acting as a critical cofactor in synthesizing all CYP450 enzymes and hormones, vitamin D, and melatonin, as well as in the methylation of proteins and DNA [69,70]. Additionally, Ca and Mg are ligands compete for the same Ca-sensing receptor (CaSR). The Western diet typically has a high Ca-to-Mg ratio. Supplemental Ca was popular, especially among women, but this changed significantly after the Women’s Health Initiative study [71] and similar studies published in subsequent years. As a result, the intake ratio increased from less than 2.5 to over 3.0, which is considered unphysiological [72].

A higher Ca-to-Mg ratio impairs vitamin D synthesis and displaces binding to the VDR, reducing its potential to interact with other essential elements [73]. The Ca:Mg ratio should be considered a “messenger” from the circulation to their target cells—especially those with CaSR—such as parathyroid cells, renal tubular cells, and the brain [74]. CaSR is a G-protein coupled receptor that detects extracellular Ca levels to maintain Ca homeostasis. The activation of CaSR in parathyroid cells reduces the secretion of PTH, while activating the renal CaSR promotes the urinary excretion of Ca [75].

The Ca-to-Mg (Ca:Mg) ratio is equally vital in maintaining systemic equilibrium [76]. A high Ca intake without adequate Mg can suppress PTH levels, negatively impacting bone remodeling and mineralization. An imbalanced Ca:Mg ratio can also exacerbate chronic conditions, including cardiovascular diseases, due to improper Ca deposition in arterial walls [16]. Ensuring a balanced ratio, ideally around 2:1 (Ca:Mg), is critical for optimizing the synergistic effects of these minerals on vitamin D metabolism, bone health, and overall physiological well-being [77]. Ca/Mg ratio (or higher blood Ca or lower Mg levels) worsens metabolic disorders. For example, persons with poorly controlled type 2 diabetes have a higher Ca:Mg ratio than those with better control [78]. 

### 4.3. Ca-to-Mg Ratio also Affects Non-Mineral Functions

The data suggest that Ca:Mg ratio (and Mg) are important variables that indicate glycemic control and complications. Other studies [75] have reported a high Ca:Mg ratio is associated with higher mortality in those with severe SARS-CoV-2 infections [79]. Others have reported that a high Ca:Mg ratio can be used as a biomarker of clinical outcomes for chronic disease, and its correction is beneficial [62]. High and low Ca:Mg ratios increase cardiovascular and all-cause mortality [80]. Ca/Mg ratios above 3.5 and below 1.70 are independently associated with an increased risk of chronic conditions, like cardiovascular disease, cancer, metabolic syndrome, type 2 diabetes, as well as all-cause mortality [81].

Evaluations and conclusions cannot be made on the efficacy of D_3_ supplementation without concomitant knowledge and attention to the Ca:Mg ratio in the circulation. In addition, patients consume D_3_ and Mg for many illnesses, which significantly impacts Ca:Mg ratio and associated therapies. Although it is not routine to measure Ca^2+^ and Mg^2+^, these can be relevant in certain circumstances and disorders. Furthermore, low adequate D_3_ is linked to gut dysbiosis, compromising the absorption of all these elements.

Mg is crucial for many biological activities, including hormone synthesis and release, as well as facilitating calcitriol-VDR receptor interactions [16,82]. Adequate Mg is essential for CTR interactions and can help reduce complications and mortality from post-COVID syndrome [83]. However, increasing Mg intake without addressing Ca overload may decrease PTH levels and impair Mg absorption. While lower circulating Mg levels typically stimulate PTH synthesis, Mg is required for both PTH synthesis and its release, so this may paradoxically reduce PTH levels [16]. The goal is to maintain the Ca:Mg ratio closer to 2.0, significantly enhancing enzymatic functions and improving vitamin D efficacy [16,77,84].

### 4.4. Importance of the Ca-to-Mg Ratio for Vitamin D and CTR Functions

Vitamin D is crucial for Ca and phosphorus metabolism and maintaining bone health and various physiological processes [85]. However, its effectiveness is significantly influenced by the balance between Ca and Mg (their ratio) (Ca:Mg) in the body [72,73]. Mg is a cofactor for enzymes that activate vitamin D, converting it into calcitriol [16,77]. Vitamin D and its receptor VDR function optimally at a Ca-to-Mg ratio of around 2.0 [84,86], supporting Ca absorption in the intestines and preventing calcification in soft tissues.

As with vitamin D_3_ and PTH [87,88], responses have many non-linear U- or J-shaped activities/curves, including Mg [61]. In addition, there are localized overlapping or built redundant feedback systems, such as 24-hydroxylase enzymes in the skin, to prevent excess vitamin D3 from entering the circulation [89]. This is not represented in circulatory or whole-body concentrations. Consequently, cause-and-effect relationships are not necessarily apparent: they are subtle but interconnected and complex, making and distinguishing them challenges. These interactions behave differently when the ratios, like Ca:Mg or high T4/T3 (thyroiditis) to low T4/T3 (Graves), make interactions even more complicated.

A sufficient amount of vitamin K_2_ in the circulation can mitigate this imbalance [90,91]. The disruption of this balance, particularly Mg deficiency, can impair vitamin D activation [73,77], leading to suboptimal Ca regulation and increased risks of bone disorders like osteoporosis [16]. Deficiencies in either mineral may lead to pathological conditions such as skeletal deformities, metabolic syndrome/obesity, cardiovascular diseases, and other inflammation-related disorders [16,72,91]. Individuals with a higher Ca:Mg ratio may also require circulating 25(OH)D concentrations above the recommended standard levels to obtain its biological activities, which may mimic pseudo vitamin D resistance. Therefore, it is advisable to supplement with Mg after assessing the Ca:Mg ratio before providing higher doses of vitamin D from Mg.

**Figure 3:**
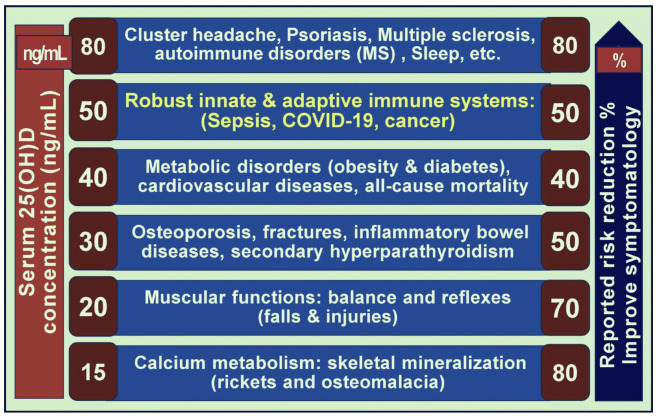
Different body systems, tissues, and diseases require varying steady-state serum 25(OH)D concentrations to prevent initiation and progression. The left side of the figure presents the minimum (average) serum 25(OH)D concentrations needed to obtain optimum clinical outcomes. In contrast, the right side depicts the percentage risk reduction for each disease entity [25(OH)D;25-hydroxyvitamin D] (modified from Wimalawansa) [94].

Increasing Ca intake in individuals with lower Ca:Mg ratios does not necessarily resolve accompanying vitamin D insufficiency. Addressing this requires D_3_ supplementation or exposure to UVB rays to stimulate endogenous production. As highlighted, tissue sensitivity can vary based on the Ca:Mg ratio (e.g., high vs. low Ca:Mg). However, there is no evidence that the physiological cut-off concentrations of circulating 25(OH)D differ by ethnicity, age, sex, or sun exposure [87,92,93]. Emerging evidence, nevertheless, suggests that optimal 25(OH)D levels vary, indicating that different tissue sensitivities and disorders require distinct (higher) serum 25(OH)D concentrations, as depicted in Figure 3.

The dose–response curves following vitamin D supplementation are curvilinear [95,96,97]. This response can become exaggerated in individuals with imbalanced Ca:Mg ratios—at high or low extremes [88]. As a result, the efficacy of D_3_ supplementation—and consequently clinical outcomes—can vary significantly unless the unbalanced Ca:Mg ratio is addressed beforehand.

### 4.5. Regulation of Ca and Phosphate Through Parathyroid Hormone

Parathyroid hormone (PTH) regulates Ca and Mg; however, Ca is the primary determinant of PTH levels, with PTH profoundly affecting ionized Ca [98]. Ca and phosphate physiology and metabolism are regulated by the hormones PTH, vitamin D, fibroblast growth factor 23 (FGF23), and calcitonin [99]. Mg is also a Ca antagonist in various ways, including functioning as a Ca channel blocker [73,100]. Understanding these interactions is crucial for comprehending the physiology of Ca regulation.

Low endogenous vitamin D_3_ levels could arise from low Mg and excess Ca, which could overwhelm Mg, leading to the downregulation of CaSRs [100]. Mg also mitigates vascular calcification and osteogenic differentiation in the presence of high Ca and phosphate in the circulation [85,92]. Commonly, low D_3_ levels result from reduced D_3_ synthesis in the skin and/or inadequate dietary intake of D_3_. In states of a high Ca:Mg ratio, excess Ca (due to high intake or increased absorption, consequently, elevated Ca in the circulation) suppresses PTH and calcitriol synthesis [73]. This suppression leads to decreased Ca and Mg absorption and reduced renal tubular resorption. Additionally, insufficient Mg intake exacerbates the synthesis of calcifediol and calcitriol.

Conversely, insufficient Ca stimulates PTH secretion at a low Ca:Mg ratio. Increasing Ca intake under such conditions further suppresses PTH and calcitriol synthesis [98]. It also inhibits Mg absorption and resorption, particularly in individuals already deficient in D_3_ due to inadequate sun exposure and/or low dietary or supplemental D_3_ intake [100]. Furthermore, elevated 25(OH)D levels (e.g., >40 ng/mL) may still be associated with a higher Ca:Mg ratio. Increasing Mg intake could lower 25(OH)D concentrations while improving the Ca:Mg balance [73]. Therefore, clinicians and researchers must remain aware of these delicate balances and interactions.

Most human hormone synthesis and release processes require physiological Mg concentrations in tissue [99]. While tissue Mg concentrations are important, no routine laboratory tests measure such levels. However, these processes can be impaired when the Ca:Mg ratio is unphysiological [93]. Therefore, the influence of Ca:Mg should be considered in specific vitamin D-resistant syndromes before initiating pharmacological vitamin D therapies [92,101]. Additionally, hypovitaminosis D disrupts the intestinal microbiome, negatively affecting mineral absorption and maintaining mineral balance. Ca and phosphate are essential for human physiology, including skeletal mineralization and neuromuscular function [98]. Some of these are discussed in the next section.

### 4.6. Importance of the Ca-to-Mg Ratio in Non-Mineral Disorders

At lower serum 25(OH)D concentrations, particularly below 20 ng/mL, circulatory PTH concentrations exhibit an exponential inverse relationship with vitamin D status [102]. A similar relationship exists between the Ca:Mg ratio and serum 25(OH)D levels, indirectly reflecting bioavailable PTH in circulation [103]. For instance, low blood Ca levels (hypocalcemia with low Ca:Mg ratio) contribute to secondary hyperparathyroidism, whereas Mg deficiency (hypomagnesemia with higher Ca:Mg ratio) can manifest as idiopathic hypoparathyroidism [81,104]. A thorough understanding of these relationships enables clinicians to manage Ca metabolism disorders and parathyroid dysfunctions better, potentially preventing unnecessary parathyroidectomy surgeries.

Ca and Mg generally compete and have opposed actions [104]. The Western diets are rich in Ca and low in Mg. Consequently, many have higher ratios than the optimal [81]. Asian and South American diets are generally lower in Ca than Western diets [103], thus generally having a lower Ca:Mg ratio, except for those who regularly consume hard water (primarily groundwater) containing high Ca salts [105,106]. In the latter group, due to very high Ca: Mg ratios (I.e., above 4.0), those who have sustained dehydration and also indulged in daily alcohol for several years develop Ca carbonate crystallization in renal tubules and tissues [107,108], causing unusual chronic renal failure affecting in tropical regions. In the presence of fluoride, these nanocrystals become stable and gradually grow [108,109]. This used to be called CKD of unknown etiology but is now remaned as CKD of crystalloid-tubular nephropathy (CKD-CTN) [107,108].

Before stage IIIB, the disease is reversible by drinking plenty of portable water. REF This is another illustration where investigating Ca: Mg ratios can help diagnose and manage such patients without drastic and expensive therapies [110]. In those with low Mg-derived high Ca:Mg ratios, Mg supplementation is the treatment of choice [110]. Most affected are farmers; thus, they are exposed to plenty of sunlight in the tropics. As a result, there is no reason to supplement them with vitamin D. In some, the 25(OH)D defects can be corrected with Mg supplements. In these situations, a more accurate picture reflecting actual ratios is obtained by expressing Ca and Mg in mmol/L (mM concentrations) than mg/L.

## 5. Phosphate and Human Health

Phosphate is another essential mineral found in both natural and processed foods. It is absorbed in the intestines, with any excess filtered by the kidneys and excreted in the urine [111]. Phosphate is crucial for various cellular processes, including ATP production, intracellular signal transduction, bone mineralization, cell membrane formation, and DNA and RNA synthesis [112,113]. Phosphate homeostasis is regulated by a complex interplay of several organs and hormones, including PTH, FGF23, klotho, and vitamin D [111]. High dietary phosphate intake can precipitate diseases such as type 2 diabetes [114].

Ca and phosphorus are involved in multiple physiological activities that affect most body systems [85]. Consequently, abnormalities in Ca and phosphorus metabolism lead to several pathological conditions, including skeletal- and cardiovascular-related disorders and premature mortality, such as renal failure [92,98]. The Recommended Dietary Allowance (RDA) for phosphorus is approximately 700 mg/day [115]. The European Food Safety Authority (EFSA) set an Adequate Intake of 550 mg/day for healthy adults, including those during pregnancy and lactation [115].

The Nordic Nutrition Recommendations (NNRs) from 2012 endorsed a slightly higher recommended intake (RI) of 600 mg/day for adults [116]. Most healthy individuals in North America consume nearly twice as much phosphate as the RDA [113]. Dietary phosphate overload is a health risk, especially for low-income populations, who often consume more inexpensive processed foods and phosphate-containing fizzy drinks containing inorganic phosphate additives [113,117]. These inorganic additives are absorbed more readily than organic phosphates in natural foods [115], leading to disorders [114].

### 5.1. Phosphate Interactions with Other Minerals

Pre-clinical and clinical research has shown that chronic dietary phosphorus excess has toxic effects, leading to adverse health outcomes [85,118]. Improved food labeling regulations and nutritional education are necessary to help individuals make informed dietary choices and reduce the risks associated with high phosphate intake [112,113]. Elevated phosphate levels have been linked to an increased risk of metabolic syndrome [98,118]. Mg is known to counteract the harmful effects of excess phosphate [98,119]. Sustained high phosphate levels trigger inflammatory responses that contribute to vascular stiffness (as in hypertension) [112], impair insulin sensitivity, and disrupt lipid metabolism (e.g., raising low-density lipoprotein levels) [117,118].

High phosphate levels decrease pancreatic insulin production, increasing the risk of type 2 diabetes and cardiometabolic diseases [112,117]. These findings emphasize the importance of proper phosphorus management in maintaining overall metabolic health [118]. In the absence of interventions, those with high phosphate diets and/or renal impairment are likely to develop phosphate toxicity [98,113], which can affect metabolic, cardiovascular, and renal health, leading to premature death and increasing the risk of tumor formation [117,119].

### 5.2. Causes of Hyperphosphatemia

Hyperphosphatemia is characterized by elevated levels of phosphate in the blood, typically exceeding 4.5 mg/dL in adults [120]. It is often associated with impaired renal dysfunction—impaired renal filtration leads to phosphate accumulation [113]. Other causes include excessive dietary phosphate intake, hypoparathyroidism, and using phosphate-containing medications [113,117]. Elevated phosphate levels disrupt the delicate balance of mineral metabolism, leading to complications such as vascular calcification, secondary hyperparathyroidism, and bone disorders [121]. Chronic hyperphosphatemia has been implicated in increased cardiovascular morbidity and mortality, particularly in individuals with chronic kidney disease (CKD), and increased mortality [120].

### 5.3. Treatment of Hyperphosphatemia

Treating hyperphosphatemia involves maintaining good hydration, combining dietary modifications and pharmacological interventions, and addressing underlying conditions [122]. Dietary phosphate restriction focuses on reducing the intake of phosphate-rich foods, mainly processed foods with inorganic phosphate additives. Phosphate binders, such as Ca acetate, sevelamer, or lanthanum carbonate, are commonly prescribed to bind dietary phosphate in the gastrointestinal tract, preventing absorption. In severe cases, particularly in individuals with advanced CKD [107,120], dialysis may be required to remove excess phosphate from the bloodstream [113,123]. Monitoring serum phosphate levels and Ca and parathyroid hormone levels is essential to guide treatment and prevent complications [121].

### 5.4. Causes of Hypophosphatemia

Hypophosphatemia is characterized by abnormally low levels of phosphate in the blood, typically below 2.5 mg/dL in adults [124]. Insufficient phosphate can disrupt physiological processes, including energy production, cell signaling, and bone mineralization [122]. The causes of hypophosphatemia include malnutrition, chronic alcohol use, vitamin D deficiency, prolonged use of antacids, refeeding syndrome, and certain medical conditions like hyperparathyroidism or Fanconi syndrome [113,121]. Symptoms can range from mild fatigue and muscle weakness to severe complications, such as respiratory failure, hemolysis, rhabdomyolysis, and impaired neurological function, depending on the severity and duration of the phosphate deficiency [124].

### 5.5. Treatment of Hypophosphatemia

The treatment of hypophosphatemia focuses on addressing the underlying cause and restoring normal phosphate levels [124]. Mild cases can often be managed with dietary modifications, such as increasing the intake of phosphate-rich foods like dairy products, nuts, and meats. Oral phosphate supplements, such as potassium phosphate or sodium phosphate, are commonly prescribed for moderate to severe cases or when dietary intake is insufficient [121]. In cases of severe hypophosphatemia with symptomatic manifestations, intravenous phosphate administration may be necessary [113]. Monitoring serum phosphate, Ca, and renal function during treatment is crucial to prevent tissue complications such as hyperphosphatemia or Ca–phosphate precipitation [121,124].

## 6. Additional Functions of Common Minerals

Minerals are crucial for maintaining various physiological functions in the human body. Essential minerals such as Ca, Mg, and phosphorus contribute to bone health, muscle function, and nerve transmission [85]. Ca, for example, is vital for bone strength and proper muscle contraction, whereas Mg supports enzymatic reactions and energy production [125]. Phosphorus is a key component of DNA, RNA, and ATP and plays a fundamental role in cellular energy metabolism.

Trace minerals, including iron, zinc, and selenium, are equally important for overall health. Iron is essential for oxygen transport in the blood through hemoglobin [126,127], while zinc supports immune function, wound healing [128], and DNA synthesis [85]. Selenium is a powerful antioxidant that protects cells from oxidative stress and supports thyroid function. A balanced intake of these minerals is critical to prevent deficiencies and ensure optimal health and well-being [125].

### 6.1. Effects of Untreated Mineral Deficiencies

Untreated mineral deficiencies can have serious long-term effects on human health. Mineral deficiencies during childhood and adolescence can significantly impact growth and development [129]. Clinically, Ca and vitamin D deficiencies can lead to poor bone mineralization, resulting in rickets in children and osteoporosis later in life. Similarly, iron deficiency can cause cognitive impairments and developmental delays in children [130,131].

Iodine deficiency during pregnancy and early childhood can result in irreversible brain damage and intellectual disabilities [129]. Numerous meta-analyses have revealed a strong relationship between iodine status during pregnancy and neonatal and maternal outcomes [132]. Similarly, a meta-analysis of 2190 pregnant women revealed a pooled prevalence of iodine deficiency during pregnancy in Ethiopia [133]. Zinc deficiency has been associated with depression and cognitive impairment in children and adults [134]. Zinc importers and exporters control the cellular zinc balance. The zinc importer family (ZIP) allows zinc to accumulate in the cytosol, while the zinc exporter family (ZnT) transports zinc out of the cytosol [135]. When required, excess zinc is eliminated through the kidneys, skin, and intestines [128].

### 6.2. Effects of Other Trace Minerals on Human Health

Several other minerals play crucial roles in maintaining a functional immune system: zinc deficiency can impair immune cell development and function, increasing susceptibility to infections. In contrast, selenium deficiency may reduce the body’s ability to fight viral infections and increase the risk of certain tumors. A prospective, randomized, double-blind, placebo-controlled multicenter trial revealed that, compared with placebo, oral zinc supplementation decreased 30-day mortality and ICU admission rates in COVID-19 patients [136].

The antimicrobial and immune-boosting effects of zinc are thought to be partly responsible for reducing infection and inflammation-related burdens [137,138]. Iron deficiency can compromise immune responses, increasing the susceptibility of individuals to infections [139]. Iron absorption in the intestine is a coordinated process in which ascorbic acid (vitamin C) creates an acidic environment, and enteric brush border Na+/H+ exchangers generate an H+ gradient for apical Fe^2+^ (ferrous iron) absorption. This process is facilitated by divalent metal transporter 1 (DMT1). Iron is then transported across the basolateral surface of enterocytes by the iron exporter protein, ferroportin, and eventually enters the bloodstream for distribution throughout the body [140].

Long-term mineral deficiencies are associated with an increased risk of various chronic diseases. For example, Mg deficiency has been linked to cardiovascular diseases, type 2 diabetes, and osteoporosis [13,141]. Ca deficiency can lead to osteoporosis and increased fracture risk, especially in older adults. Iron deficiency anemia can cause fatigue, weakness, and, in severe cases, heart problems. Minerals play essential roles in various metabolic processes; chromium deficiency can impair glucose tolerance and insulin resistance, potentially increasing the risk of type 2 diabetes [142,143,144].

Copper deficiency may cause anemia and neutropenia, affecting oxygen transport and immune function. Both zinc and Mg inadequacy are also linked to metabolic disorders [145]. Mineral deficiencies can also impact reproductive health and pregnancy outcomes; for example, iron deficiency during pregnancy increases the risk of preterm birth and low birth weight, and zinc deficiency may contribute to complications during pregnancy and childbirth [146].

Essential trace elements, including molybdenum, cobalt, and chromium, are also important for human health. Molybdenum acts as a cofactor for enzymes involved in the metabolism of sulfur-containing amino acids [147]. It helps in the production of uric acid and involves glucose metabolism. Although its deficiency is rare in humans, it may cause headaches, seizures, visual impairments, and neurological disorders. A low risk of toxicity in humans and high consumption (10–15 mg/day) could induce gout-like symptoms and joint pain [147].

Cobalt is an essential vitamin B_12_ (cobalamin) component (Figure 1), and its deficiency can lead to anemia [148]. Chromium, however, is crucial for protein, fat, and carbohydrate metabolism, and its deficiency can cause impaired glucose tolerance. While chromium is necessary for these metabolic processes, excessive intake can result in skin diseases and liver and kidney damage and may even promote various tumors. These elements are vital for human health, thus, maintaining a healthy physiological balance, and avoiding deficiency and toxicity is crucial but can be delicate, especially for micro minerals like chromium and molybdenum [149].

### 6.3. Importance of Minerals During Pregnancy and Lactation

Minerals play crucial roles in pregnancy and lactation, and their inadequacy could significantly impact the health of both mothers and fetuses. For example, zinc is important for fetal development and milk secretion [150]. Maintaining Ca is essential for fetal growth of the musculoskeletal system and teeth [150]. Adequate Ca balance is also needed to reduce the risk of maternal hypertension and preeclampsia. Similarly, iodine is necessary for healthy brain development [151,152]. Mineral balance is important during breastfeeding, and mineral requirements increase during lactation [153,154].

**Figure 4:**
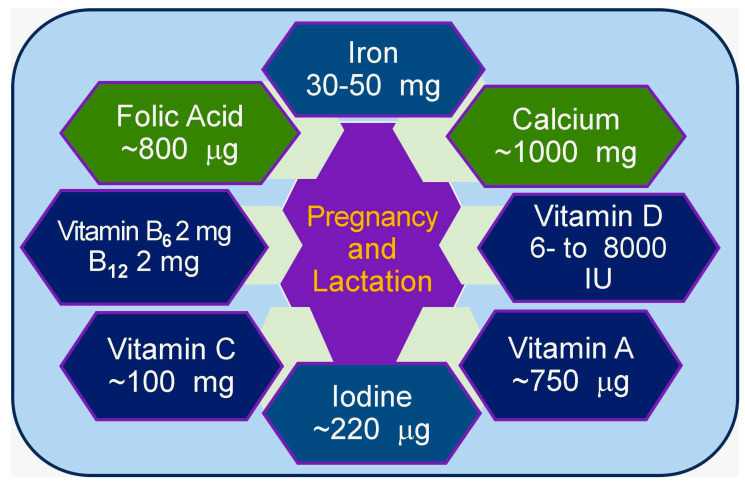
A few important nutrients with approximate total (diet plus supplements) daily requirements during pregnancy and lactation.

Higher Mg intake is recommended for fetal growth and development, especially during early lactation [37]. Adequate mineral intake during pregnancy is essential for maternal health, fetal growth and development, and pregnancy and lactation. Although increased mineral intake through diet and supplements can meet maternal nutritional needs, avoiding excessive intake is also important [152]. Personalized dietary counseling and supplemental recommendations can help pregnant women meet the minimum mineral requirements to ensure adequate and prevent excessive intake (Figure 4).

### 6.4. Abnormal Mineral Metabolism Secondary to Genetic Abnormalities

Genetic mutations can lead to mineral metabolism disorders in several ways. Many genetic mutations affect enzymes involved in mineral metabolism pathways. For example, mutations in the CYP27B1 gene, which encodes the 1α-hydroxylase enzyme, can cause vitamin D-dependent rickets type 1A by impairing the synthesis of calcitriol (the active component of vitamin D) [155]. Mutations in the CYP24A1 gene, which encodes a 24-hydroxylase gene, can lead to idiopathic infantile hypercalcemia by disrupting vitamin D degradation. Mutations can also affect receptors crucial for mineral homeostasis.

VDR gene mutations can cause vitamin D-dependent type 2 rickets, impairing the body’s response to vitamin D. CaSR gene mutations can lead to familial hypocalciuric hypercalcemia or autosomal dominant hypocalcemia [156]. Similarly, mutations in genes encoding mineral transporters can also disrupt mineral absorption or excretion, as mutations affecting sodium-phosphate co-transporters can lead to renal phosphate waste and induce skeletal deformities [157]. Genetic defects can impact hormones regulating mineral metabolism, and mutations in the PTH gene can cause hypoparathyroidism. Similarly, mutations affecting FGF23 can lead to disorders of phosphate homeostasis [158,159].

Defects in genes encoding transcription factors that regulate mineral metabolism genes can have widespread effects. For example, GATA3 mutations can cause hypoparathyroidism, deafness, and renal dysplasia syndrome [160]. Some genetic disorders affect multiple systems, including mineral metabolism. Examples include multiple endocrine neoplasia type 1 (MEN1) and autoimmune poly-endocrinopathy-candidiasis-ectodermal dystrophy (APECED) [161]. These genetic defects can result in a wide range of mineral metabolism disorders, from relatively mild imbalances to severe, life-threatening conditions. The specific effects depend on the gene involved, the nature of the mutation, and environmental factors. Understanding these genetic mechanisms is crucial for diagnosing, treating, and potentially developing gene therapies for mineral metabolism disorders [162].

### 6.5. Strategies to Reduce the Burden of Mineral Deficiencies

The safest and most cost-effective way to reduce mineral deficiency is to increase the consumption of micronutrient-rich foods [163]. There should be a combined approach addressing supply and demand by increasing the production of micronutrient-rich foods and promoting increased consumption through education and behavior change efforts. Food-based fortification strategies should be implemented by promoting small-scale vegetable and fruit gardens (home gardens), encouraging the production of small animals, poultry, and fish as sources of bioavailable micronutrients [164], and supporting efficient large-scale commercial production of fruits and vegetables [163,165]. In addition, food fortification by adding synthetic nutrients to commonly consumed staple foods and biofortification by breeding crops to increase their nutritional value should be considered [166], including food literacy [167].

Moreover, education and increasing awareness of communication techniques to promote changes in eating practices would provide long-term benefits [167]. Furthermore, community involvement by ensuring community participation, especially of women, in program design and implementation with the participation of local people in assessment, analysis, and action to increase acceptance would be beneficial. The most effective approach is likely to be a combined strategy that addresses increased production and consumption while meeting the needs of particular groups, such as children and women of childbearing age [168].

## 7. Mineral Toxicity and Human Health

Mineral toxicity occurs when the concentration of certain minerals in the body becomes abnormally high, leading to adverse health consequences. Excess sodium in the bloodstream, known as hypernatremia, can be highly harmful; the normal sodium level in plasma is 136–145 mM, and >152 mM is considered toxic. Hypernatremia can lead to confusion, seizures, coma, paralysis of the lung muscles, and death. Hypernatremia causes brain cells to shrink, leading to severe neurological effects [169].

Hyperkalemia can also be life-threatening. The normal concentrations range from 3.5 to 5.0 mM. When the concentration is high (6.3–8.0 mM), severe toxicity, including cardiac arrhythmias, cardiac arrest, and death, occurs [170]. The body usually minimizes potassium toxicity by inducing a vomiting reflex and increasing kidney function [123]. Hyperkalemia often occurs in individuals with various kidney diseases (unable to remove excess potassium) or individuals taking certain medications (potassium-sparing diuretics, ACE inhibitors) [170,171].

Iron poisoning is particularly threatening for children. Mineral toxicity is most common in U.S. children under 6 years of age [172]. Approximately 20,000 cases of accidental iron ingestion in children occur annually, with symptoms of nausea, vomiting, abdominal pain, shock, liver failure, and death in severe cases [173,174]. Iron poisoning can be acute (from a single large dose) or chronic (from long-term accumulation). The severity of iron poisoning depends on the amount of elemental iron ingested, with children being particularly vulnerable due to their smaller size [175].

Although not conclusively proven, aluminum exposure may be a risk factor for Alzheimer’s disease [176]. Other effects include nausea, skin ulcers and rashes, vomiting, diarrhea, arthritic pain, and nervous system issues (memory loss and balance problems). Cadmium exposure is often occupational but can occur through contaminated water or food [177]. Acute toxicity can induce abdominal pain, vomiting, and diarrhea, whereas chronic toxicity can induce kidney disease, lung damage, and bone weakness [178].

Several genetic disorders can increase the risk of mineral toxicity by affecting the body’s ability to regulate certain minerals properly [179]. Hemochromatosis is one of the most common genetic disorders associated with mineral toxicity. It affects approximately 1 in 10 people in the United States who carry genetic mutations, causing excessive absorption and iron accumulation in the body, leading to iron overload and toxicity if left untreated [162]. Approximately 1 million people in the U.S. have hemochromatosis [180,181].

Wilson’s disease is a rare inherited disorder of copper metabolism caused by a mutation in the ATP7B gene encoding a canalicular copper-transporting ATPase [182]. It affects approximately 1 in 30,000 people, causing copper to accumulate in the liver, brain, and other organs [183]. If not managed, it can lead to copper toxicity [182]. Copper is an essential mineral that provides antioxidant protection to the body, and its inadequacy reduces the body’s ability to cope with oxidative stress [183,184].

Menke’s disease is a rare X-linked recessive disorder affecting copper metabolism that primarily affects males. The incidence ranges from 1 in 50,000 to 1 in 250,000 individuals, causing copper deficiency in the brain and other tissues [185], but can paradoxically lead to copper accumulation in some organs. Importantly, having a genetic predisposition does not always ensure that mineral toxicity will occur. Environmental factors, diet, and overall health also play significant roles.

## 8. Discussion

Minerals are essential in food and vital for the development and physiological functions of the body. Minerals play important roles, including structural functions such as building and maintaining bones, teeth, and soft tissues (e.g., Ca, phosphorus, and Mg), as cofactors in enzymatic reactions, neural functions, and even receptor interactions (as with vitamin D) and membrane stability (Figure 1). Minerals regulate many biological processes, such as electrolytes and fluid balance (e.g., sodium, potassium, and chloride), maintaining heart rhythm, nerve responses, and signal conduction (e.g., Ca, potassium, and sodium), as well as blood clotting (e.g., Ca).

Iron is crucial for forming hemoglobin, which transports oxygen to the blood. Minerals also support immune function (e.g., zinc, copper, and selenium) and aid in metabolism and energy regulation, such as thyroid hormone production (e.g., iodine). Additionally, they are required to synthesize DNA and the function of antioxidants, such as glutathione (e.g., Mg and zinc), and breakdown proteins, carbohydrates, and cholesterol (e.g., manganese). Vital dietary sources of minerals include fruits, vegetables, whole grains, cereals, lentils, lean meats, and dairy products.

Untreated mineral deficiencies can significantly impact overall health, affecting physical growth, cognitive function, and disease susceptibility. Maintaining adequate mineral intake through a balanced diet or supervised supplementation is crucial for long-term well-being. Proper diagnosis and management of genetic conditions related to mineral metabolism can help prevent toxicity risks. Balancing mineral intake is essential for optimal health outcomes.

## 9. Conclusions

Mineral dysregulation contributes to a substantial economic burden on societies, particularly in developing countries with high prevalence rates. These inadequacies reduce work productivity and increase healthcare costs, leading to significant economic losses associated with chronic diseases. Much of this economic toll could be avoided through proper prevention and treatment of mineral deficiencies or toxicity. Excessive supplementation should be avoided, and occupational exposure should be safe to prevent mineral toxicity. While minerals are vital for health, maintaining proper balance is essential; consultation with a healthcare professional is recommended if mineral toxicity is suspected. In summary, minerals play essential roles in maintaining the functions of most body systems, and maintaining an adequate balance is vital for health and well-being.

## Figures and Tables

**Table 1:** Common minerals, their sources, physiological functions, and potential toxicity in the human body.

Minerals	Sources	Main Physiological Functions	Potential Human Toxicity
**Boron**	Fruits (avocados, raisins, peaches, apples, grapes, oranges, bananas); vegetables and legumes (broccoli, potatoes, carrots, celery); nuts and seeds (peanuts, almonds, Brazil nuts and hazelnuts)	It affects the metabolism of steroid hormones (estrogen, testosterone, and vitamin D), improves cognitive performance in older adults, and improves antioxidant activity and wound healing.	Skin reactions (rashes and dermatitis) and neurological symptoms (headaches, restlessness, and convulsions) affect fertility and male reproductive organs
**Calcium**	Milk and milk products; canned fish with bones (salmon and sardines); fortified tofu and soy beverage; greens (broccoli and mustard greens); legumes	Important for bone and teeth health; second messenger.	Kidney stones and nephrocalcinosis, abnormal heart rhythms, and vascular calcification
**Chloride**	Table salt, soy sauce, and processed foods	Maintaining pH levels in the body (particularly in the blood) is essential for producing hydrochloric acid (in the stomach), facilitating the transmission of nerve impulses, and maintaining proper fluid balance and blood pressure.	Impair sodium or potassium metabolism
**Chromium**	Whole grains (bread and cereals, oatmeal, and barley); seafood (mussels, oysters, and shrimp); fruits (apples, bananas, and grapes); lean meats (beef, turkey, and chicken); vegetables (broccoli, green beans, potatoes, and asparagus)	Blood sugar regulation is achieved by improving insulin sensitivity; protein metabolism is achieved through the breakdown and absorption of proteins; and fatty acid and cholesterol synthesis is stimulated.	Skin issues (contact dermatitis, skin ulcers, and sensitization), liver and kidney damage; increased risk of lung cancer
**Cobalt**	Fish and shellfish (oysters, clams, and mussels), meat (mainly liver and kidney), milk and dairy products, legumes, beans and nuts	Essential components of vitamin B12 (cobalamin), erythropoiesis, cofactor for methyl malonyl-CoA mutase, and methionine synthase.	Chronic exposure may lead to asthma-like symptoms, toxic cardiomyopathy, cognitive decline, and polycythemia
**Copper**	Legumes, nuts and seeds, whole grains, organ meats, and drinking water	Many enzymes are needed for iron metabolism.	Liver damage, kidney failure, neurological effects (mood changes, depression, anxiety, irritability, and difficulty focusing), hematological effects (hemolytic anemia)
**Fluoride**	Beverages (black tea and coffee, grape juice, chocolate, and almond milk); fruits (grapes and raisins, apples, strawberries, bananas, peaches, watermelon, and cherries); vegetables (spinach, potatoes, carrots, and asparagus); seafood (shrimp, crab, and oysters)	Preventing and reversing dental caries by strengthening tooth enamel, contributing to the mineralization and strength of skeletal tissues, increases the stability and crystallinity of bone apatite structures.	Dental fluorosis (white chalky opacities on tooth enamel, brownish discoloration or pigmentation and pitting of tooth); skeletal fluorosis (increased bone density and decreased elasticity, joint pain and decreased mobility, and increased risk of fractures)
**Iodine**	Seafood, foods grown in iodine-rich soil, iodized salt, bread, and dairy products	Iodine is present in the thyroid hormone, which helps regulate growth, development, and metabolism.	Thyroid dysfunction (hypothyroidism, hyperthyroidism, thyroiditis, or increased risk of thyroid cancer); neurological effects (delirium, seizures, and stupor)
**Iron**	Organ meats, red meats, fish, poultry, shellfish (especially clams), egg yolks, legumes, dried fruits, dark leafy greens, and iron-enriched breads and cereals	Part of a molecule (hemoglobin) found in red blood cells that carries oxygen in the body needed for energy metabolism.	Liver damage (chronic liver disease, cirrhosis, and increased risk of hepatocellular carcinoma); cardiac effects (heart failure and arrhythmias); neurological effects (potential acceleration of neurodegenerative diseases)
**Magnesium**	Nuts and seeds, legumes, leafy green vegetables, seafood, and chocolate	Maintain skeletal system and immune system health.	Gastrointestinal effects (diarrhea and abdominal discomfort); cardiovascular effects (hypotension, bradycardia, and heart blocks); neuromuscular effects (muscle weakness and paralysis)
**Manganese**	Nuts and seeds (hazelnuts, pecans, and pine nuts); legumes (chickpeas, soybeans, and lentils); shellfish (mussels, oysters, and clams)	A key component of the antioxidant enzyme superoxide dismutase (SOD); it plays a role in blood clotting and hemostasis; acts as a cofactor for various enzymes.	Cognitive impairment, increased susceptibility to respiratory tract infections, and slurred speech
**Molybdenum**	Legumes (black-eyed peas, lima beans, lentils, and pinto beans); whole grains (oats, barley, and brown rice); dairy products (milk, yogurt, and cheese); vegetables (spinach, potatoes, and asparagus)	The enzyme cofactor for xanthine oxidase and aldehyde oxidase plays a role in the liver’s phase I and II detoxification pathways.	Joint pain and gout-like symptoms, anemia, and neurological effects (seizures and hallucinations)
**Phosphorus**	Meat, fish, poultry, eggs, and milk	Important for healthy bones and teeth; maintains acid-base balance.	Cardiovascular calcification, impaired renal functions, and dysregulation in bone metabolism
**Potassium**	Meats, milk, fresh fruits and vegetables, whole grains, and legumes	Needed for proper fluid balance, nerve transmission, and muscle contraction.	Cardiovascular effects (palpitations, arrhythmias, and potential heart attack); neurological effects (fatigue, headache, delirium, or seizures); muscle-related effects (weakness, pain, and in severe cases, and paralysis)
**Selenium**	Meat, seafood, and grains	A key component of antioxidant enzymes, particularly glutathione peroxidases, is important for male fertility and spermatogenesis in regulating thyroid hormones. Selenium-containing enzymes help make DNA and protect against cell damage.	Dermatitis, alopecia, nail discoloration, peripheral neuropathy, decreased cognitive function, and cardiovascular issues (tachycardia and palpitations)
**Silicon**	Legumes (soybeans, tofu, and red lentils); nuts and seeds (almonds, peanuts, and sunflower seeds); whole grains (oats, barley, and brown rice)	It plays a role in maintaining the structural integrity and elasticity of skin, hair, and nails, crucial for synthesizing and stabilizing collagen.	Excessive exposure can cause silicosis (a progressive and irreversible lung disease); exposure to silica dust can also increase the risk of lung cancer, chronic obstructive pulmonary disease (COPD), and tuberculosis; long-term exposure increases the risk of autoimmune diseases.
**Sodium**	Table salt, soy sauce, and processed foods	Plays a key role in regulating blood pressure by influencing blood volume and vascular tone; critical for the conduction of nerve impulses, allowing proper communication between nerve cells; essential for normal muscle contraction and relaxation; involved in the transport of various nutrients across cell membranes, including glucose, amino acids, and phosphate.	Neurological effects (confusion, seizures, coma, potential cerebrovascular damage, thirst and dehydration, muscle weakness, and pain)
**Zinc**	Meats, fish, poultry, whole grains, and vegetables	They are needed for making protein and genetic material and have a role in taste perception, wound healing, normal fetal development, sperm production, normal growth and sexual maturation, and immune system health.	Copper deficiency (resulting in anemia and neutropenia); impaired immune function; and neurological effects (lethargy, dizziness, and, in severe cases, convulsions)

## Data Availability

Data included in the article are referenced in the article.
